# Supplementary material for: Prevalence of substance use and associated factors among secondary school adolescents in Kilimanjaro region, northern Tanzania
Source: PLoS One. 2022 Sep 2;17(9):e0274102. doi: 10.1371/journal.pone.0274102 (PMC9439258; doi:10.1371/journal.pone.0274102)
Supplement: Attachment — Submitted filename: PONE-D-21-31212R2.pdf [file pone.0274102.s002.pdf]

Dear Dr. Mavura,

Thank you for revising your manuscript up to this point! However, you have not been responsive to the suggestion of the reviewer, i.e., "My remaining point is that the discussion should focus on studies conducted in Africa and other low- and middle-income countries. ... the authors should consider revising paragraph 3 of the discussion section that makes reference to studies conducted in the United States." Nevertheless, you still make reference to studies in the United States where there has been enormous studies on substance use among youth and it will be under-reporting to refer to the United States in the Discussion. Indeed, I agree with the reviewer that the focus should be on studies conducted in African countries, not elsewhere.

While it is true that "there are limited studies about the association between adolescent age and substance use in SSA", extensive studies have been conducted on the use of substances such as tobacco using the GYTS data etc. The substances considered in this study are alcohol, cigarette smoking, marijuana, khat, and recreational drugs (cocaine, heroin); therefore, the discussion should relate to studies in SSA on any of these substances. GYTS studies on the association between age and initiation of tobacco use/cigarette smoking exist. As such, I will encourage the authors to review the literature and use the results to address the issue raised by the reviewer (including paragraphs 2 and 3). The GSHS does not capture the landscape of substance use among adolescents in SSA, so you should look outside that for such studies.

Thank you,
Prof. Hadii
M. Mamudu Guest Editor
