# Supplementary material for: Prevalence of substance use and associated factors among secondary school adolescents in Kilimanjaro region, northern Tanzania
Source: PLoS One. 2022 Sep 2;17(9):e0274102. doi: 10.1371/journal.pone.0274102 (PMC9439258; doi:10.1371/journal.pone.0274102)
Supplement: Attachment — Submitted filename: Response to Reviewers1.docx [file pone.0274102.s001.docx]

P. O. Box 2240,

Moshi – Tanzania.

**Journal requirements:**

When submitting your revision, we need you to address these additional requirements.

1. Please review your reference list to ensure that it is complete and correct. If you have cited papers that have been retracted, please include the rationale for doing so in the manuscript text, or remove these references and replace them with relevant current references. Any changes to the reference list should be mentioned in the rebuttal letter that accompanies your revised manuscript. If you need to cite a retracted article, indicate the article’s retracted status in the References list and also include a citation and full reference for the retraction notice.

Response: Thank you for the comment, we have reviewed the references list and none of the references have been retracted.

**Reviewer comments:**

1. My remaining point is that the discussion should focus on studies conducted in Africa and other low- and middle-income countries. As such, the authors should consider revising paragraph 3 of the discussion section that makes reference to studies conducted in the United States.

Response: Thank you for the good review and comment. We have edited paragraph three which states as follows: “However, previous studies in Zambia [27] and the United States[28, 29] found high substance use practice among adolescents 15 years and above. Likewise, analysis of GSHS data from six Asian low and middle-income countries revealed a higher risk of substance use (alcohol and smoking) among older compared to younger adolescents [30]. However, there are limited studies about the association between adolescent age and substance use in SSA.”

References

27.	Siziya S, Muula AS, Besa C, Babaniyi O, Songolo P, Kankiza N, et al. Cannabis use and its socio-demographic correlates among in-school adolescents in Zambia. Ital J Pediatr. 2013;39: 1–5. doi:10.1186/1824-7288-39-13

28.	Jones CM, Clayton HB, Deputy NP, Roehler DR, Ko JY. Prescription Opioid Misuse and Use of Alcohol and Other Substances Among High School Students — Youth Risk Behavior Survey , United States , 2019. 2020;69: 38–46.

29.	Creamer MR, Jones SE, Gentzke AS, Jamal A, King BA. Tobacco Product Use Among High School Students — Youth Risk Data Source. 2020;69: 56–63.
