# Supplementary material for: Prevalence of substance use and associated factors among secondary school adolescents in Kilimanjaro region, northern Tanzania
Source: PLoS One. 2022 Sep 2;17(9):e0274102. doi: 10.1371/journal.pone.0274102 (PMC9439258; doi:10.1371/journal.pone.0274102)
Supplement: Attachment — Submitted filename: Response to Reviewers1.docx [file pone.0274102.s003.docx]

P. O. Box 2240,

Moshi – Tanzania.

16^th^ August 2022.

**Journal requirements:**

When submitting your revision, we need you to address these additional requirements.

1. Please review your reference list to ensure that it is complete and correct. If you have cited papers that have been retracted, please include the rationale for doing so in the manuscript text, or remove these references and replace them with relevant current references. Any changes to the reference list should be mentioned in the rebuttal letter that accompanies your revised manuscript. If you need to cite a retracted article, indicate the article’s retracted status in the References list and also include a citation and full reference for the retraction notice.

Response: Thank you for the comment, I have reviewed my reference list and none of the references have been retracted

**Reviewer comments:**

1. My remaining point is that the discussion should focus on studies conducted in Africa and other low- and middle-income countries.

Response: Thank you for the good review and comment, we have edited paragraph we have edited paragraphs that did include cite studies from united states and other high income countries and focused more on sub-Saharan Africa ,low and middle income countries. The edited paragraph are will viewed on the document with track changes.
